# Whole‐genome resequencing reveals recent divergence of geographic populations of the dung beetle *Phelotrupes auratus* with color variation

**DOI:** 10.1002/ece3.9765

**Published:** 2023-01-24

**Authors:** Yoshifumi Araki, Teiji Sota

**Affiliations:** ^1^ Department of Zoology, Graduate School of Science Kyoto University Kyoto Japan

**Keywords:** demographic history, human impact, population divergence, whole‐genome resequencing

## Abstract

Knowledge of population divergence history is key to understanding organism diversification mechanisms. The geotrupid dung beetle *Phelotrupes auratus*, which inhabits montane forests and exhibits three color forms (red, green, and indigo), diverged into five local populations (west/red, south/green, south/indigo, south/red, and east/red) in the Kinki District of Honshu, Japan, based on the combined interpretation of genetic cluster and color‐form data. Here, we estimated the demographic histories of these local populations using the newly assembled draft genome sequence of *P. auratus* and whole‐genome resequencing data obtained from each local population. Using coalescent simulation analysis, we estimated *P. auratus* population divergences at ca. 3800, 2100, 600, and 200 years ago, with no substantial gene flow between diverged populations, implying the existence of persistent barriers to gene flow. Notably, the last two divergence events led to three local populations with different color forms. The initial divergence may have been affected by climatic cooling around that time, and the last three divergence events may have been associated with the increasing impact of human activities. Both climatic cooling and increasing human activity may have caused habitat fragmentation and a reduction in the numbers of large mammals supplying food (dung) for *P. auratus*, thereby promoting the decline, segregation, and divergence of local populations. Our research demonstrates that geographic population divergence in an insect with conspicuous differences in traits such as body color may have occurred rapidly under the influence of human activity.

## Graphical abstract

*Phelotrupes auratus* , the geotrupid dung beetle with three color forms diverged into five geographic population groups based on the combined interpretation of genetic cluster and color‐form data. We estimated the demographic histories of these population groups using the newly assembled draft genome sequence of *P. auratus* and whole‐genome resequencing data obtained from the groups.

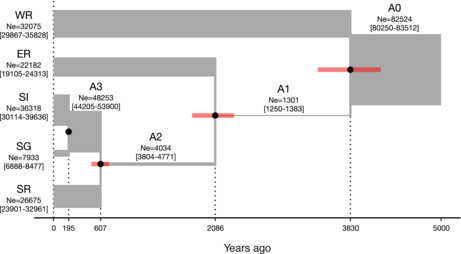

## 1 INTRODUCTION

Knowledge of the history of population divergence is crucial to our understanding of organisms' diversification mechanisms. The evolutionary histories of wild organisms have been shaped largely by past climatic and geographic events. For example, environmental changes that occurred during the last glacial period strongly influenced species' distribution patterns. The combined interpretation of past climatic and geographic events and molecular population genetic data has provided a powerful means to elucidate the evolutionary history of organisms over relatively long time scales such as the Quaternary (Hewitt, 2000, 2004). In recent years, the impact of human activities on the evolution of organisms has gradually become clear. Human‐induced environmental changes have affected the evolution of adaptive traits in many organisms (Sullivan et al., 2017) and have led to the decline and extinction of wild populations (Ceballos et al., 2015). During the early to mid‐Holocene, humans began to practice agriculture and pastoralism, rapidly increasing their population size and impact on the environment (Boivin et al., 2016). It is thus important to consider the possibility that evolutionary changes in the populations of wild organisms have been influenced by not only climatic and geographic events but also human activities.

Genetic variation between local populations is determined by the balance of the homogenizing effect of gene flow and the diversifying effect of local adaptation (Endler, 1977). Geographic variation in adaptive traits often reflects genetic differences among populations due to restricted gene flow. Among many adaptive traits, coloration and pigment patterns are inferred as inter or intraspecific signals that have been used to define populations in studies of evolutionary processes (Curran et al., 2020; Hoyal Cuthill & Charleston, 2015; McLean & Stuart‐Fox, 2014; Mullen et al., 2009; Stankowski et al., 2017). Recently, the development of high‐throughput sequencing technology has enabled the acquisition of large amounts of fine‐scale sequence variation data that provide powerful clues about the processes underlying population divergence through whole‐genome resequencing (Ellegren, 2014) or reduced‐representation approaches such as restriction site‐associated DNA sequencing (RAD‐seq; Baird et al., 2008). Currently, great efforts are being made to estimate past demographic changes and evolutionary events that have occurred in various wildlife populations based on large amounts of genomic data (Excoffier et al., 2013; Li & Durbin, 2011; Schiffels & Wang, 2020; Terhorst et al., 2017; Zhou et al., 2020).

**FIGURE 1:**
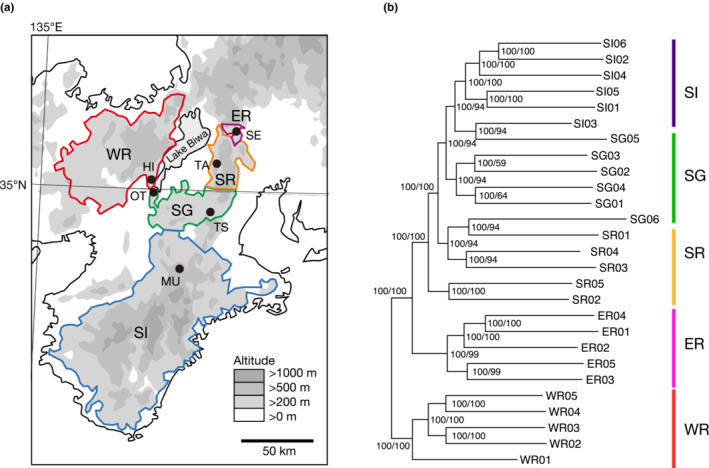
(a) Distribution areas and sampling sites of *P. auratus* in the Kinki District of Honshu, Japan. Distribution areas (encircled with different colors): red, west/red; purple, east/red; blue, south/indigo; green, south/green; and orange, south/red. For sampling sites (closed circles), see Table 1. (b) Maximum‐likelihood tree of *P. auratus* individuals from the five local populations. Node support values indicated on branches are SH‐aLRT and UF‐bootstrap values (percentages).

Here, we focused on the geotrupid dung beetle *Phelotrupes auratus* (Motschulsky). It exhibits notable variation in its structural metallic body coloration, which is provided by a simple multilayer reflector structure (Akamine, Ishikawa, et al., 2011) and is categorized as red, green, or indigo based on the peak wavelength on the elytral reflectance spectrum (Akamine et al., 2008; Araki & Sota, 2021). *Phelotrupes auratus* is a diurnal beetle that inhabits montane forests; its food resource is the dung of large wild mammals, especially sika deer (Tsukamoto et al., 2014). In the Kinki District of Honshu, the main island of Japan, the three color forms occur separately in different geographic areas (Akamine, Maekawa, & Kon, 2011; Figure 1a); however, the mechanism by which this variation formed and has been maintained remains unclear. Our previous population structure analysis using RAD markers (Araki & Sota, 2021) showed that *P. auratus* populations in the Kinki District diverged into five geographic groups defined by combinations of three color forms and three genetic clusters—west/red (WR), east/red (ER), south/indigo (SI), south/green (SG), and south/red (SR)—and that geographic color variation was maintained by hybrid transition zones with steep genetic clines (i.e., barriers to gene flow) or transition zones without genetic differentiation. In the latter zone type, we inferred the action of strong selection on different body colors in different regions. However, as the function of body color in *P. auratus* is unclear, the inference that color differentiation is maintained solely by selection despite frequent gene flow was questionable. Obscure genetic differences between sister populations may have been caused by very recent population divergence, even if they are separated by barriers to gene flow. Therefore, the lack of detection of genetic differentiation in our previous study may be attributable to the low resolution of the RAD‐seq data and/or the methodology of our population clustering analysis. Analysis using a larger genomic dataset, such as that provided by whole‐genome resequencing, may provide new insights into geographic color diversification in *P. auratus*.

In this study, to construct robust demographic history models and estimate the dynamics of effective population sizes, gene flow, and divergence times among populations, we constructed a de novo draft genome of *P. auratus* and analyzed genome‐wide single‐nucleotide polymorphism (SNP) data obtained by whole‐genome resequencing of individuals sampled from five local populations. We found that the five local populations diverged recently—within several thousand years, under the influence of human activity—and that no substantial gene flow had occurred among the diverged populations. Our findings reveal an unexpected influence of human activity on population differentiation through the divergence of a conspicuous trait in this insect species.

## 2 MATERIALS AND METHODS

### 2.1 Sampling and individual coloration data

Between 2019 and 2020, *P*. *auratus* adults were collected using traps baited with horse dung at six locations around Lake Biwa in the Kinki District, Honshu, Japan (Figure 1a). To obtain five or more individuals from each of the five local populations of *P*. *auratus* identified in the region, one sampling site was selected for each population based on our previous data; SG was sampled at two locations to obtain sufficient samples. A total of 27 beetles were collected (Table 1) and fixed in 99% ethanol; one beetle collected from SI was immersed in RNAlater solution for DNA and RNA sampling and then stored at −30°C until DNA/RNA extraction. As in our previous study (Araki & Sota, 2021), we quantified the elytral colors of individuals using a spectrometer (USB2000 + UV–VIS‐ES; Ocean Optics) to measure the reflection spectra of the elytral surfaces of dry specimens in a dark room and classified the color forms as indigo (<525 nm), green (525–600 nm), or red (>600 nm; Araki & Sota, 2021).

**TABLE 1:** *Phelotrupes auratus* individuals from which genomic DNA were extracted in this study

Individual ID	Color: λmax(α) (nm)	Population group	Population; locality name; latitude (°N), longitude (°E)
WR01	631.81	West/Red (WR) ” ” ” ”	HI; Mt. Hiei, Kyoto; 35.0640, 135.8224 ” ” ” ”
WR02	632.14		
WR03	620.05		
WR04	660.79		
WR05	642.28		
ER01	665.09	East/Red (ER) ” ” ” ”	SE; Sekigahara, Gifu; 35.2627, 136.5234 ” ” ” ”
ER02	677.06		
ER03	664.75		
ER04	645.28		
ER05	673.21		
SI01	483.45	South/Indigo (SI) ” ” ” ” ”	MU; Murou, Nara; 34.5391, 136.0285 ” ” ” ” ”
SI02	494.14		
SI03^a^	526.91		
SI04	517.73		
SI05	505.14		
SI06	486.39		
SG01	552.90	South/Green (SG) ” ” ” ” ”	OT; Mt. Otowa, Kyoto; 34.9768, 135.8530 ” ” ”
SG02	579.57		
SG03	638.50		
SG04	589.58		
SG05	596.04		TS; Tsuge, Mie; 34.8542, 136.2750 ”
SG06	516.80		
SR01	598.64	South/Red (SR) ” ” ” ”	TA; Mt. Takatori, Shiga; 35.2804, 136.3768 ” ” ” ”
SR02	578.78		
SR03	633.15		
SR04	632.81		
SR05	612.19		

^a^ Used for constructing the reference genome sequence.

### 2.2 Genomic DNA extraction and sequencing

Total genomic DNA was extracted from the flight muscle and testes using a Genomic‐tip 20/G kit (QIAGEN), following the manufacturer's protocol. The quality of DNA samples was assessed with Qubit (Thermo Fischer Scientific), NanoPhotometer (Implen), and 2200 TapeStation (Agilent) instruments. For genome assembly, the genomic DNA of an SI individual (SI03) was sent to GeneBay, Inc. (Yokohama) for library construction and long‐read sequencing using the ONT PromethION system (Oxford Nanopore Technologies). Short‐read sequencing was performed with 150‐bp paired‐end reads in three runs on an Illumina HiSeq X Ten sequencer (Illumina) at Macrogen Japan. For genome assembly, a single sample (SI03) was sequenced using one lane of the HiSeq X Ten sequencer. For resequencing, 10 samples from the SI and ER groups and 15 samples from the WR, SG, and SR groups were separately sequenced using two lanes of the HiSeq X Ten sequencer. Library construction for SI and ER samples was performed at Macrogen Japan and that for WR, SG, and SR samples was performed at Kyoto University using the NEB Next Ultra II FS DNA Library Prep Kit for Illumina (E7805S; New England Biolabs). Adapters on raw reads were trimmed using Porechop (v0.2.4, https://github.com/rrwick/Porechop) for ONT PromethION long reads and fastp (Chen et al., 2018) for Illumina HiSeq X short reads.

### 2.3 Transcriptomic RNA extraction and sequencing

For gene prediction on the reference *P. auratus* genome, we obtained mRNA sequence data from an adult SI specimen (not included in Table 1) fixed in RNAlater solution. Total RNA was extracted using a Monarch Total RNA Miniprep Kit (New England Biolabs Japan), following the manufacturer's protocol. Stranded paired‐end 150‐bp library construction and sequencing were conducted using GENEWIZ. Adapters on raw reads were trimmed using fastp.

### 2.4 Genome assembly for reference genome construction

Genome size was estimated with Illumina short reads using GenomeScope (Vurture et al., 2017, http://qb.cshl.edu/genomescope/) based on the result of the 31‐mer count obtained with Jellyfish v2.3.0 (Marçais & Kingsford, 2011). We de novo assembled a draft genome of *P. auratus* (individual SI03) at the scaffold level using Illumina HiSeq X short reads and ONT PromethION long reads by a hybrid assembly procedure using MaSuRCA 3.4.2 (Zimin et al., 2013). The constructed draft genome was polished with racon 1.4.13 (Vaser et al., 2017) and Pilon 1.23 (Walker et al., 2014) and then curated using Purge Haplotigs 1.0.4 (Roach et al., 2018). Using BUSCO v5.2.5 (Simão et al., 2015), the quality of the curated draft genome was checked against the “endopterygota_odb10” database (updated on 10 September 2020, containing 2124 single‐copy ortholog genes); masked tandem repeats were detected using RepeatModeler 2.0.1 (Flynn et al., 2020) and RepeatMasker 4.1.1 (Smit et al., 2015). Gene prediction was conducted with the BRAKER v2.1.4 pipeline (Barnett, Garrison, Quinlan, Str̈mberg, & Marth, 2011; Brůna et al., 2021; Hoff et al., 2016; Hoff et al., 2019; Lomsadze et al., 2014; Stanke et al., 2008; Stanke et al., 2006) using the draft genome and mapped RNA‐seq data. RNA‐seq mapping was performed with hisat2 v2.2.1 (Kim et al., 2019) and samtools.

### 2.5 Read mapping and variant calling

Short reads were mapped to the draft genome with bwa‐mem 0.7.17 (Li & Durbin, 2009) and sorted with picard SortSam v2.25.7 (https://broadinstitute.github.io/picard/). The average read depth was 16.02, excluding SI03 (read depth, 154.11). SNP calling was conducted for each sample using bcftools 1.12 (Li et al., 2009); genotype likelihoods were computed using the mpileup command, and variants were called using the call command. Based on the average read depth of each sample, generated by samtools 1.13 (Li et al., 2009), we collected SNPs with Phred quality scores >10 and depths between two‐thirds (or 10 if the average depth was <15×) and twice the average depth, and removed indels. Called variants were merged into a single VCF file with bcftools merge, then filtered with maf >0.05, max missing <0.15, −m = 2, and −M = 2 to remove rare substitutions, missing abundant sites, and multiple substitution sites.

### 2.6 Phylogenetic and genetic population structure analyses

A phylogenetic tree was constructed using all the obtained SNP data and the maximum‐likelihood method in IQ‐TREE 2.1.2 (Minh et al., 2020) with 1000 UF‐bootstrap iterations (Hoang et al., 2018) and 1000 bootstrap replicates for the SH‐like approximate likelihood ratio test (SH‐aLRT; Guindon et al., 2010). The best‐fitting substitution model was estimated using ModelFinder (Kalyaanamoorthy et al., 2017) with an ascertainment bias correction model (Lewis, 2001); as a result, PMB + F + ASC + R4 was selected. In the optimal topology, five individuals of WR were assigned to the outgroup based on the results of our previous study (Araki & Sota, 2021), although we did not assume the monophyly of WR individuals prior to the phylogenetic analysis. We also performed population genetic structural analysis using the ADMIXTURE v1.3.0 software (Alexander et al., 2009) with the number of ancestral clusters, *K*, ranging from 2 to 5 and principal component analyses using the pcaMethods package in the R software (Stacklies et al., 2007) based on the SNPs to assess differences in the genetic compositions of individuals among the populations.

### 2.7 Demographic history

We used SMC++ v1.15.2 (Terhorst et al., 2017) with the number of spline knots set to 25 to estimate the demographic history of each population and population divergence times using the SNP data, excluding SNPs on the scaffolds <100 kb in length. Previously published genome‐wide mutation rates in dipteran, lepidopteran, and hymenopteran insects are similar, ranging from 2.8 e‐09 to 3.4 e‐09 per base per haploid genome per generation (Keightley et al., 2014, 2015; Liu et al., 2017; Yang et al., 2015). Because data on the mutation rate and generation time of *P. auratus* are lacking, we used the rate of *Heliconius melpomene* (2.9 × 10^−9^ per generation; Keightley et al., 2015) as the mutation rate in the coalescent simulations and 2 years as the generation time (Tateno et al., 2020). Estimations were conducted 20 times with 20 iterations, and the median and 95% confidence intervals (CIs) of the estimated size history were calculated using the nonparametric bootstrap method. In addition, we estimated demographic parameters using fastsimcoal2.7 (Excoffier et al., 2013), based on the topology estimated by IQ‐TREE2. The coalescent simulation requires constraints on the divergence time of one or more nodes of the tree. In the absence of prior information on the divergence times of *P. auratus* populations, we used the results of the SMC++ analysis to determine the upper constraints on divergence time and effective population size. Recent secondary contacts and past gene flow were designed for contiguous (or ancestral) populations in the current geographic distribution. To simplify the simulations, we assumed that each population had a constant size with no growth after population divergence; to disregard recombination rates by minimizing linkage disequilibrium among the SNPs used in the analysis (Excoffier et al., 2013), LD‐based SNP pruning was conducted using the SNP data and PLINK 1.90 (Purcell et al., 2007) with the command ‐‐indep‐pairwise 10,000 10 0.2. The model was run 100 times with the following settings: number of coalescent simulations in each cycle, 100,000; expectation/conditional maximization (ECM) cycles, 40; minimum ECM cycles, 10; minimum observed site frequency spectrum (SFS) entry count, 10; and removal of monomorphic sites for SFS. The 95% CIs of the parameters were calculated using a nonparametric bootstrap method.

## 3 RESULTS

### 3.1 Genome sequence of *P. auratus*

The genome size of *P. auratus* estimated by the GenomeScope analysis was 680,802,890 bp with 2% heterozygosity. The assembled draft genome contained 9727 scaffolds with a total length of 867,702,560 bp (scaffold N50, 148,001 bp) and was thus longer than the estimated size, possibly due to insufficient removal of haplotigs. Gene prediction resulted in the detection of 30,643 transcripts in 28,887 protein‐coding regions from the draft genome; 30,252 (98.72%) transcripts were annotated in the BLAST search. BUSCO score quality assessment revealed 1810 (85.2%) complete single‐copy, 226 (10.6%) duplicated, 30 (1.4%) fragmented, and 58 (2.8%) missing BUSCOs. Variant calling against the draft genome for all resequencing data (*n* = 27) resulted in the assembly of a dataset with 9,184,739 SNPs in 8838 scaffolds (rate of missing SNP data, <0.01%). We used this dataset in the subsequent analyses with and without SNP pruning for LD. After LD pruning, 247,662 SNPs remained in the dataset (rate of missing SNP data, 3.51%).

### 3.2 Phylogenetic and population genetic structural analyses

**FIGURE 2:**
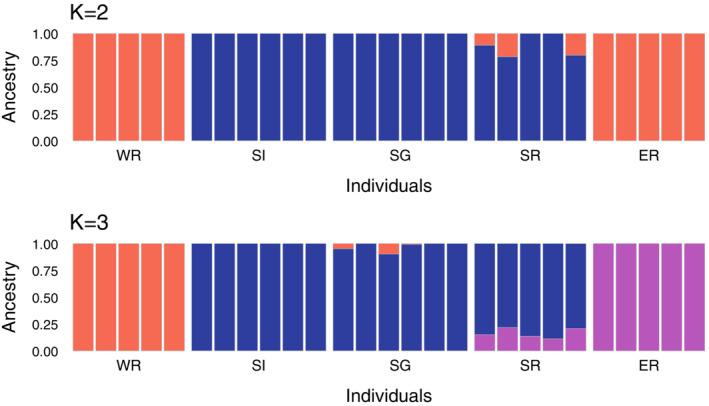
Genetic population structure of *P. auratus* individuals at each site. Orange, ancestry 1; blue, ancestry 2; and purple, ancestry 3. The optimal number of clusters (*K*) was found to be 2.

**FIGURE 3:**
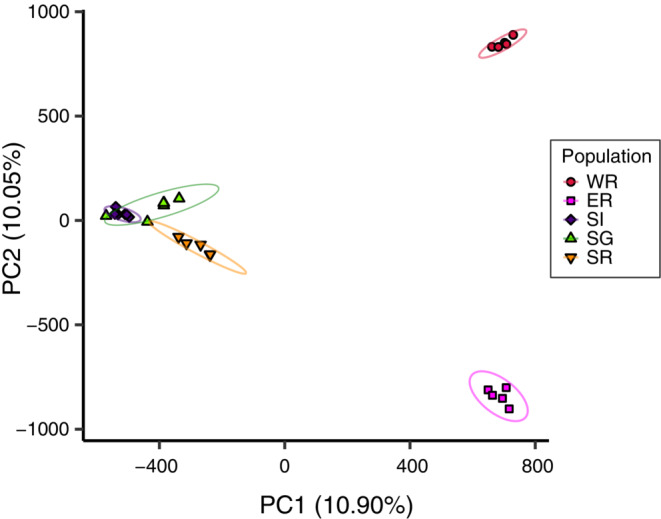
Principal components 1 (PC1) and 2 (PC2) scores of *P. auratus* individuals determined from principal component analyses of single‐nucleotide polymorphisms. Local population: red circles, west/red; magenta squares, east/red; blue diamonds, south/indigo; green triangles, south/green; and inverted pink triangles, south/red. Ellipses, 95% confidence ellipses of PC scores for each population.

The phylogenetic analysis revealed that individuals from WR, ER, and the southern populations (SI, SG, and SR) formed different clades. Within the southern populations, one SG individual (SG05) was included in the clade of SI individuals, and one SG individual (SG06) was included in the clade of SR individuals. The population structural analysis (Figure 2) showed that the optimal number of ancestral groups (*K*) was 2 based on cross‐validation error values (0.75 at *K* = 2; 0.88 at *K* = 3; 1.04 at *K* = 4; 1.23 at *K* = 5); individuals were divided into one group including the WR and ER populations and another including the SI, SG, and SR populations. However, individuals from WR and ER were clearly discriminated at *K* = 3 (Figure 2). The first two principal components showed genetic differences among the three groups: WR, ER, and the southern population group (SI, SG, and SR) (proportions of variance explained: PC1, 10.90%; PC2, 10.05%; Figure 3). Although there was little genetic divergence among the southern populations, the genetic divergence between SR and SI + SG individuals was also shown by the PC1–PC2 plot. These population genetic structure results were similar to our previous RAD‐seq results (Araki & Sota, 2021).

### 3.3 Demographic history

**FIGURE 4:**
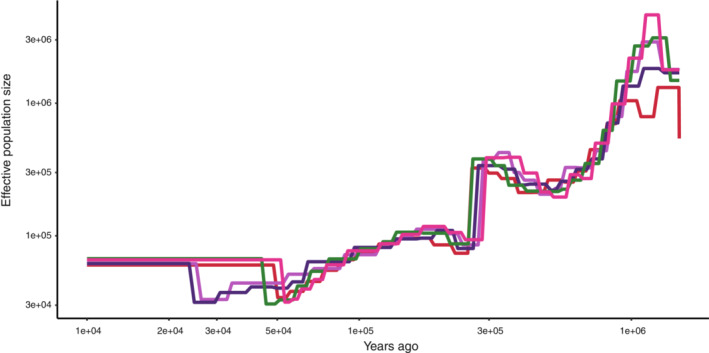
Historical changes in effective population sizes, inferred by SMC++. Line colors represent local populations: red, west/red; purple, east/red; blue, south/indigo; green, south/green; and orange, south/red.

We first estimated the demographic history of each population and the divergence times between pairs of populations based on the SNP data using SMC++. The temporal changes in the effective population size were consistently similar among all populations until approximately 50,000 years ago (Figure 4). The estimation of divergence times showed that WR and ER diverged from the lineage of the southern populations (SI, SG, and SR) approximately 27,000–47,000 years ago and a subsequent divergence among populations occurred approximately within the last 9000 years (Table S1).

**FIGURE 5:**
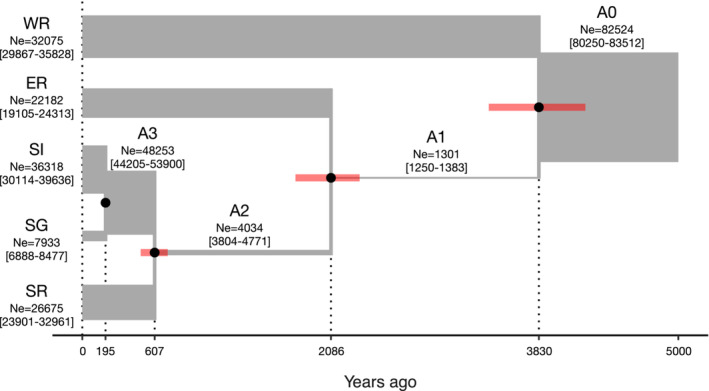
Demographic history of *P. auratus* populations inferred by coalescent simulation using fastsimcoal2 during the past 5000 years. Horizontal bar widths indicate effective population sizes, *N*_e_ (median and 95% confidence intervals are indicated by numerals); black circles and red vertical bars indicate medians and 95% confidence intervals, respectively, of divergence times.

We performed a coalescent simulation with fastsimcoal2 to reconstruct the population divergence history with gene flow using a model with the upper limit of the effective population size set to 100,000 and that of the first divergence event from the most recent common ancestor (MRCA) set to 30,000 generations (60,000 years) ago, based on the results of the SMC++ analysis. Parameter estimation using the demographic model revealed the following demographic histories. WR and A1 (MRCA of ER, SI, SG, and SR) diverged 3830 (95% CI, 3410–4220) years ago, ER and A2 (MRCA of SI, SG, and SR) diverged 2086 (95% CI, 1788–2326) years ago, SR and A3 (MRCA of SI and SG) diverged 607 (95% CI, 490–716) years ago, and SI and SG diverged 195 (95% CI, 174–208) years ago (Figure 5). For the effective population size, the A1 population experienced a severe bottleneck; the A2 and ER populations showed slight increases; and further population increases occurred in SI, SG, and SR (Figure 5, Table S2). Although we assumed continuous gene flow between populations, the estimated effective number of migrants (*N*_e_ *m*) was much lower than unity (10^−10^ to 10^−7^) for all cases (Figure 5, Table S3), suggesting a lack of substantial gene flow (Slatkin, 1987).

## 4 DISCUSSION

### 4.1 Demographic histories of the geographic populations

Our demographic analysis revealed the history of divergence of the five geographic populations of *P. auratus*. The SMC++ results revealed long‐term historical changes in the effective population sizes. The similarity in the patterns of demographic change among populations may reflect the low degree of genetic differentiation among populations until recently (Figure 4). Application of the split function in SMC++ revealed that WR diverged from the other populations first, approximately 49,000 years ago (Table S1). This result is consistent with the results of population structural analysis (Figure 2) and our previous finding that WR diverged deeply from the other populations (Araki & Sota, 2021). However, the divergence times estimated by SMC++ were much older than those estimated by fastsimcoal2. This difference may have resulted from methodological differences between the two programs. First, SMC++ completely ignores gene flow and estimates the demography of each population independently, and the estimated coalescent times are based solely on divergence in population size between populations (Terhorst et al., 2017); by contrast, fastsimcoal2 can consider gene flow between populations in demographic estimations (Excoffier et al., 2013). Second, SMC++ can use linkage information among SNPs in demographic estimations to consider linkage disequilibrium (LD) and allow maximal use of SNP data (Terhorst et al., 2017), whereas fastsimcoal2 generally requires minimizing linkage disequilibrium in the SNP data by pruning linked SNPs to reduce the complexity of models and render the simulation feasible (Excoffier et al., 2013). These methodological differences can result in discrepancies in the estimation of demographic history. Regarding the demographic estimation, sequential Markovian coalescent (SMC)‐based methods such as SMC++ are reliable for estimating relatively ancient demographic histories, whereas SFS‐based methods such as fastsimcoal2 are reliable for estimating recent demographic histories (Patton et al., 2019). Accordingly, the divergence time estimation using SMC++, which is based on changes in estimated population sizes, may be inaccurate for recent population divergences. Further analyses are needed to understand the differences in divergence times estimated using different approaches.

The coalescent simulation conducted with fastsimcoal2 and the incorporation of gene flow in the model revealed that no substantial gene flow among the five populations had occurred since their divergence (Figure 5), suggesting that the geographic color variation of *P. auratus* is maintained mainly by barriers to gene flow. In our previous study, we inferred the occurrence of frequent gene flow among the three southern populations (SR, SG, and SI) and the presence of hybrid zones between WR and SG and between ER and SR (Araki & Sota, 2021), and the genetic similarity among southern populations was also supported by the population structural analysis conducted in this study. However, the fastsimcoal2 analysis revealed that the divergence of the southern populations occurred within the past 600 years, implying that an insufficient number of unique genetic variations had accumulated in each population to detect divergence between local populations through population clustering analysis, despite the establishment of geographic isolation.

### 4.2 Factors restricting gene flow

Although *P. auratus* has dispersal potential by means of flight, they are confined to mountain forest areas and do not occur in wide open lands between lowland forests. The distribution of *P. auratus* among separate mountain areas in the Kinki District reflects this habitat selection pattern (Figure 1a). Forest fragmentation can also lead to a decline in the supply of food resources for *P. auratus*, as forests are also the main habitat of large mammals in the Japanese islands. Therefore, forest fragmentation would have affected population divergence in this species.

The confinement of *P. auratus* to forest areas may also be related to the high risk of predation during flight outside of forests. The metallic coloration of beetles may be effective for predator avoidance when they fly among light and dark forest patches (Schultz, 1986). However, when beetles fly out into open land, they are easily tracked by birds. Thus, migration between patchy forests is likely to be difficult. The cost of subsocial behavior on parents with larval nests may also restrict the time allowed for dispersal by flight. Large dung beetles that construct deep nests and lay few, large eggs are expected to have low dispersal rates (Hanski & Cambefort, 2014). This trend is likely to occur in *P. auratus*, as females build nests at depths of 50–100 cm and lay only one large egg in each nest (Tateno et al., 2020); this relatively sedentary lifestyle may limit opportunities for dispersal and migration.

### 4.3 Factors influencing population divergence

Our fastsimcoal2 analysis showed that *P. auratus* populations in the Kinki District diverged about 3800 years ago and more recently (Figure 5). Although the Holocene climate has been relatively stable and warm (Petit et al., 1999), minor local climatic changes (± 2°C) have occurred several times (Seppä et al., 2009; Wang et al., 2011). In Eurasia, marked climate cooling occurred 4200–4000 years ago, which may have caused the decline of several human civilizations, including the Jomon population in Japan (Yasuda et al., 2004). Japan also experienced climatic cooling 3800–3600 years ago (Kawahata et al., 2009; Yasuda et al., 2004). These cooling events approximately 4200–3600 years ago may have affected the population divergence of *P. auratus* approximately 3800 years ago. The time of the second divergence event (2000 years ago) coincides roughly with the beginning of the rapid increase in the human population in Japan due to the introduction of rice farming, which provided a stable food resource (Biraben, 2006; Schiffels & Durbin, 2014). Thus, population divergence 2000 years ago and more recently may have been caused by habitat fragmentation due to declines in the populations of large mammals, such as deer, under the influence of human activities such as hunting and exploitation. The increased human population size may have reduced wild animal populations and fragmentated habitat (Watanabe et al., 2019).

Declines in mammal populations due to hunting may have led to declines in the species richness and abundance of dung beetles (Andresen & Laurance, 2007). *Phelotrupes auratus* is thought to depend largely on sika deer dung, due to its overlapping distribution with sika deer (Toda & Akei, 2003). In addition, *P. auratus* likely has limited dispersal propensity due to its reproductive ecology. Thus, reduction and fragmentation of the sika deer population may have strongly affected the *P. auratus* population. Although *P. auratus* can also use dung produced by other large mammals as food resources, archeological records for the Jomon period (1200–2400 years ago) and documents mentioning local products from the mid‐Edo era (1730 s) have shown that sika deer and other large mammals such as wild boars had reduced their distribution areas by the 18th century (Tsujino et al., 2010). A genetic structural analysis also suggested that the sika deer population in Japan has experienced habitat fragmentation and bottlenecks due to human activity since the mid‐19th century (Goodman et al., 2001). To test our hypothesis that human activity has impacted large mammal and dung beetle populations, it is necessary to study the demographic history of large mammals that provide dung food for *P. auratus* with sufficient temporal and spatial resolution.

### 4.4 Divergence of color morphs among local populations

Although we detected marked divergence of local populations with different coloration within short periods in this study, it remains uncertain whether different structural coloration has adaptive importance (Araki & Sota, 2021). In a previous study, a Müllerian mimicry hypothesis was proposed based on the similarity of geographic color variation patterns between *P. auratus* and a sympatric congener, *Phelotrupes laevistriatus* (Watanabe et al., 2002). However, carcasses of the these beetles are often found in the feces of mammals such as raccoon dogs (Matsuyama et al., 2006) and chickens have been observed to feed on them without any problems (Kochi, 2001), implying that the *Phelotrupes* beetles are not unpalatable. As mentioned previously, the metallic coloration of *P. auratus* may be effective for avoiding bird predation in forests, where they fly among light and dark patches (Schultz, 1986). However, color differences may not be important in this predator avoidance strategy. If differences in the structural coloration of *P. auratus* are not important, then color differences may have been fixed by chance. A reduction in the effective population size of *P. auratus* was occurring when the indigo and green forms diverged (Figure 5), which may indicate that this color divergence was driven by genetic drift.

## 5 CONCLUSION

In this study, we conducted a detailed genomic analysis of genetic information from five local populations of *P. auratus*. Our analyses revealed that geographic color variation in *P. auratus* is of very recent origin and is maintained by restricted gene flow. The recent divergence of these populations may have been influenced by increased human activity in the past few thousand years, providing an interesting example of the indirect impact of human activity on trait divergence in wild insect populations. Our study implies the need for additional research conducted with a population genomics approach to reveal the historical relationships between the trophic levels, namely large mammals and dung beetles. Future studies of the demographic history of sika deer and other large mammals using genomic data will be essential to our understanding of the process of recent *P. auratus* population differentiation.

## AUTHOR CONTRIBUTIONS

**Yoshifumi Araki:** Conceptualization (equal); data curation (lead); formal analysis (lead); investigation (lead); writing – original draft (lead). **Teiji Sota:** Conceptualization (equal); funding acquisition (lead); project administration (lead); supervision (lead); writing – review and editing (lead).

## CONFLICT OF INTEREST

The authors declare that there is no conflict of interest.

## Supporting information


Table S1:
Table S2:
Table S3:

## Data Availability

The raw sequence reads are available from the DDBJ Read Archive (DRA) of the Data Bank of Japan (DDBJ) (BioProject; PRJDB10365; BioSample ID: SAMD00510209‐ SAMD00510222). The draft genome of *Phelotrupes auratus* is available at Dryad (https://doi.org/10.5061/dryad.z8w9ghxh2).
