# Supplementary material for: Whole‐genome resequencing reveals recent divergence of geographic populations of the dung beetle Phelotrupes auratus with color variation
Source: Ecol Evol. 2023 Jan 24;13(1):e9765. doi: 10.1002/ece3.9765 (PMC9873872; doi:10.1002/ece3.9765)
Supplement: Supplementary file 1 — Table S1: Table S2: Table S3: [file ECE3-13-e9765-s001.docx]

**Whole-genome resequencing reveals recent divergence of geographic populations of the dung beetle *Phelotrupes auratus* with color variation**

Authors

Yoshifumi Araki and Teiji Sota

Affiliations

Department of Zoology, Graduate School of Science, Kyoto University, Sakyo, Kyoto, 606-8502, Japan

Correspondence

Yoshifumi Araki and Teiji Sota, Department of Zoology, Graduate School of Science, Kyoto University, Sakyo, Kyoto, 606-8502, Japan.

Email: y.araki@terra.zool.kyoto-u.ac.jp; sota.teiji.4e@ kyoto-u.ac.jp

Table S1. Divergence time between populations of *P. auratus* estimated by SMC++.

| Population pair | Divergence time  Median (95% CI), years ago |  |
| --- | --- | --- |
| WR – ER | 48,901 (46,539–54,277) |  |
| WR – SI | 41,873 (38,513–46,732) |  |
| WR – SG | 33,656 (30,689–34,941) |  |
| WR – SR | 40,927 (39,536–43,338) |  |
| ER – SI | 35,282 (33,900–38,504) | |
| ER – SG | 33,499 (32,201–34,810) |  |
| ER – SR | 27,446 (27,061–27,992) |  |
| SI – SG | 6,753 (6,338–6,936) |  |
| SI – SR | 8,950 (8,022–9,364) |  |
| SG – SR | 5,742 (2,454–9,004) |  |

Table S2. Median and 95% CI of estimated effective population size.

| Population | Median | (95% CI) |
| --- | --- | --- |
| west/red | 32,075 | (29,867–35,828) |
| east/red | 22,182 | (19,105–24,313) |
| south/indigo | 36,318 | (30,114–39,636) |
| south/green | 7,933 | (6,888–8,477) |
| south/red | 26,675 | (23,901–32,961) |
| A0 | 82,524 | (80,250–83,512) |
| A1 | 1,301 | (1,250–1,383) |
| A2 | 4,034 | (3,864–4,771) |
| A3 | 48,253 | (44,205–53,900) |

Table S3. Median and 95% CI of estimated effective number of migrants per generation (*N*_e_*m*).

| Migration | From | To | Period | *N*_e_*m* (95% CI) |
| --- | --- | --- | --- | --- |
| NeM123 | SI | SG | Present–T1 | 2.00e-08 (1.71e-09–4.62e-07) |
| NeM132 | SG | SI | Present–T1 | 1.55e-08 (1.53e-09–2.43e-07) |
| NeM134 | SG | SR | Present–T1 | 6.30e-09 (7.33e-10–1.19e-07) |
| NeM143 | SR | SG | Present–T1 | 4.52e-08 (7.81e-09–7.97e-07) |
| NeM224 | A3 | SR | T1-T2 | 1.10e-07 (5.54e-09–9.63e-07) |
| NeM242 | SR | A3 | T1-T2 | 4.69e-09 (6.25e-10–3.42e-08) |
